# The effect of a preconception care outreach strategy: the Healthy Pregnancy 4 All study

**DOI:** 10.1186/s12913-019-3882-y

**Published:** 2019-01-23

**Authors:** Meertien K. Sijpkens, Sabine F. van Voorst, Lieke C. de Jong-Potjer, Semiha Denktaş, Arnoud P. Verhoeff, Loes C. M. Bertens, Ageeth N. Rosman, Eric A. P. Steegers

**Affiliations:** 1 Department of Obstetrics and Gynecology, Division of Obstetrics and Prenatal Medicine, Erasmus University Medical Center, Erasmus MC, P.O. Box 2040, 3000 CA Rotterdam, The Netherlands; 2 Department of Social and Behavioral Sciences, Erasmus University College, Erasmus University Rotterdam, Nieuwemarkt 1A, 3011 HP Rotterdam, The Netherlands; 3 Department of Psychology, Education and Child Studies, Erasmus University Rotterdam, Burgemeester Oudlaan 50, 3062 PA Rotterdam, The Netherlands; 4 Department of Sociology, University of Amsterdam, P.O. Box 15508, 1001 NA Amsterdam, The Netherlands; 5 Department of Epidemiology, Health Promotion and Care Innovation, Public Health Service Amsterdam, Nieuwe Achtergracht 100, 1018 WT Amsterdam, The Netherlands

**Keywords:** Preconception care, Health care utilization, Primary care, Implementation, Health behavior

## Abstract

**Background:**

Preconception care has been acknowledged as an intervention to reduce perinatal mortality and morbidity. However, utilization of preconception care is low because of low awareness of availability and benefits of the service. An outreach strategy was employed to promote uptake of preconception care consultations. Its effect on the uptake of preconception care consultations was evaluated within the Healthy Pregnancy 4 All study.

**Methods:**

We conducted a community-based intervention study. The outreach strategy for preconception care consultations included four approaches: (1) letters from municipal health services; (2) letters from general practitioners; (3) information leaflets by preventive child healthcare services and (4) encouragement by peer health educators. The target population was set as women aged 18 to 41 years in 14 Dutch municipalities with relatively high perinatal morbidity and mortality rates. We evaluated the effect of the outreach strategy by analyzing uptake of preconception care consultations between February 2013 and December 2014. Registration data of applications for preconception care as well as participant questionnaires were obtained for analysis.

**Results:**

The outreach strategy led to 587 applications for preconception care consultations. The majority of applications (*n* = 424; 72%) were prompted by the invitation letters (132,129) from the municipalities and general practitioners. The effect of the municipal letter seemed to fade out after 3 months.

**Conclusions:**

Outreach strategies amongst the general population promote uptake of preconception care consultations, although on a small scale and with a temporary effect.

**Electronic supplementary material:**

The online version of this article (10.1186/s12913-019-3882-y) contains supplementary material, which is available to authorized users.

## Background

Early pregnancy has been acknowledged as critical for the outcome of pregnancy and health later in life [1, 2]. It is therefore important to minimize risk factors for adverse embryonic growth and development even before conception. Preconception care (PCC) has been advocated to identify and modify relevant risks (e.g. biomedical, behavioral, and social risks) to a woman’s health and pregnancy outcome before conception [1, 3].

PCC’s potential has increasingly gained attention in the Netherlands. Recognition that Dutch perinatal mortality rates are higher than rates in other comparable European countries has placed PCC both on the political and professional agenda [4, 5]. This has resulted in governmental advisory reports, guidelines and tools for professionals [6, 7]. However, despite the evidence in favor of implementing PCC, it is still an uncommon form of care in the Netherlands as well as in many other countries [8, 9]. It is challenging to deliver PCC at a population level and different complementary approaches are likely to be necessary [10, 11]. An important challenging factor seems to be low awareness about preconception health and PCC among women [12, 13]. Since the prevalence of preconception risk factors is high [14, 15], this requires educating women or couples about preconception health and PCC. Integration into routine care could be one strategy, but this would not be sufficient to reach the target population, because there is no system for routine preventive care as seen in some other countries. We hypothesized that by reaching out to women of reproductive age to educate them about PCC, we could increase the uptake of PCC among women considering getting pregnant. As such, we could reach the majority of the target population, since most pregnancies in the Netherlands are planned.

In the multi-municipal Healthy Pregnancy 4 All (HP4All) PCC study, general practitioners (GPs) and midwives were incentivized to deliver PCC, whilst a community based four-pronged outreach strategy was employed to promote uptake of PCC by women who are planning to become pregnant [16, 17]. The rationale of the HP4All PCC study has been described more extensively elsewhere [17]. The main objective of this study was to evaluate the effect of the HP4All PCC outreach strategy in terms of uptake of PCC consultations.

## Methods

### Setting

The study was conducted within the HP4All program. This program started in 2011 and was financed by the Dutch Ministry of Health, Welfare and Sports. It included preventive interventions in the preconception period (PCC) and antenatal period (new approach to antenatal risk-assessment) with the ultimate aim to improve pregnancy outcomes and reduce perinatal health inequalities in the Netherlands [16]. To attain maximum effect, the interventions were delivered in high-risk neighborhoods (zip code areas) in 14 selected municipalities with perinatal mortality and morbidity rates above the national average. The selection process of the municipalities has been described elsewhere [16]. Five municipalities were clustered as they were relatively small and belonged to the same province. As a result, we refer to a total of ten municipalities in this study. In these municipalities, the target population of the study is defined as women of reproductive age (i.e. 18–41 years). Therefore, the target population was 165,615 women. The annual number of pregnancies of about 11,058 women reflects the potential number of candidates for PCC.

### Study design

**Fig. 1:**
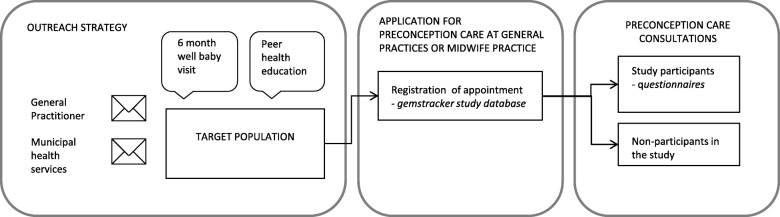
Flowchart Healthy Pregnancy 4 All preconception care strategy and study

The HP4All PCC study was designed as a community-based PCC intervention study and included the identification of a prospective cohort of participating women who utilized the PCC services (see Fig. 1). To draft this study we used Andersen’s model of healthcare utilization as our theoretical framework (see Additional file 1) [17]. The model explains how the outreach strategy would likely interact with the target population via predisposing, enabling and need characteristics, which ultimately may lead to the uptake of PCC consultations.

#### Intervention; the PCC outreach strategy

The outreach strategy for PCC had four main components targeting women aged between 18 and 41 years: 1) Participating municipalities were requested to send a mailing with information about the possibility for PCC consultations to all women in the target age range residing in the selected neighborhoods; 2) Participating GPs were requested to send a similar invitation letter to all of their female patients aged 18 to 41 years; 3) Preventive child healthcare services, responsible for monitoring and promoting optimal growth and development of children aged 0–4 years, were asked to inform parents with invitation leaflets at the regular 6 months well-baby visit; 4) Lastly, a training was offered to instruct peer health educators to organize preconception health education sessions for the target group of women aged 18–41 years considering getting pregnant. Peer health educators would then encourage this group to visit a PCC service. All four approaches were based on promising results of earlier Dutch studies using comparable approaches [18–21]. The four approaches were seen as complementary parts of one outreach strategy. They all included information on what PCC entails (personal advice, answers on fertility and health questions, good preparation for pregnancy), as well as information on when to apply for PCC (when considering pregnancy) and how to make an appointment at a PCC service (see Additional file 2). The HP4All PCC services consisted of two consultations offered by GP and midwifery practices in the designated neighborhoods. These professionals received training to provide PCC in accordance with the study protocol and the national guideline [7, 17].

#### Cohort study of women who utilized the PCC services

All women aged from 18 up to and including 41 years who made an appointment for a PCC consultation at a study practice were eligible to participate in the cohort study. Eligibility was independent of the outreach approach that preceded PCC application. When women gave permission to be approached for the study, a member of the research team contacted them by telephone to counsel about participation in the cohort study. The study had the following exclusions criteria: not attending the PCC appointment, not wishing to get pregnant, and not speaking Dutch, English, Turkish, Polish or Arabic.

### Data collection

#### Intervention; the PCC outreach strategy

Outreach strategies were implemented when GPs and midwives were ready to deliver PCC within the HP4All study. Directly after the first outreach approach of a strategy was implemented, the GPs and midwives registered all applications for PCC in an online database used for the study (Gemstracker; Generic Medical Survey Tracking System). They registered the date of the appointment and which outreach approaches women indicated as the trigger to make the appointment. We obtained information on the total number of women aged from 18 up to and including 41 years that resided in the selected neighborhoods from municipal registries. The total number of births of women in the respective zip codes was obtained from Perined (http://perined.nl). Perined is a national perinatal registry and collects information on more than 97% of all deliveries in the Netherlands from midwives, gynecologists and pediatricians.

#### Cohort study of women who utilized the PCC services

If women who applied for PCC agreed to participate in the cohort study, they were asked to fill in a questionnaire (on paper or via an internet link) before the consultation. The questionnaire contained questions regarding determinants from our model for PCC utilization (see Additional files 1 and 3). These determinants included socio-demographic characteristics, as well as details on the medical and obstetric history, lifestyle behavior, attitude and knowledge with regards to preconception health and PCC. The first municipality started data collection in February 2013 and the last municipality started in February 2014. Participants were enrolled until December 31st 2014.

### Outcomes and data-analysis

#### Intervention; the PCC outreach strategy

We determined the effect of the outreach strategy for PCC by analyzing the uptake of PCC consultations in total and per component of the outreach strategy. This was expressed in absolute numbers of women who applied for PCC and, if possible, as percentages of the number of women approached and of the average annual number of deliveries in the targeted areas. We also illustrated the duration of the ‘outreach effect’ of the municipal letters specifically by plotting a timeline showing the PCC appointments as a result of letters sent by each municipality.

#### Cohort study of women who utilized the PCC services

We reflected upon the outreach of the strategy by analyzing the data collected from the questionnaires filled in by the participants of the cohort study, who had utilized the PCC services. In line with the framework used for PCC utilization (see Additional file 1), we analyzed data on different characteristics: 1) socio-demographic characteristics; 2) barriers, beliefs and knowledge with regards to preconception health and PCC; and 3) the need and motivation for PCC, which included pregnancy and preconception health characteristics (i.e. medical and obstetric history and lifestyle behavior). These characteristics were described either continuously (mean or median with standard deviation (SD) or interquartile range (IQR)), or descriptively (percentages), as appropriate.

## Results

### The PCC outreach strategy

#### PCC outreach strategy implementation

An overview of the implementation of the outreach strategy components is provided in Table 1. The adoption of the components differed by municipality (2nd column). The potential outreach in all municipalities together was set as the total number of women aged 18–41 years residing in these areas, which consisted of 165,615 women. The outreach strategy reached the majority of these women with at least one approach (3th column). The last column of Table 1 provides the uptake per outreach approach, given as the actual number of women who made an appointment and reported these specific outreach approaches.

**Table 1:** Overview of the outreach of the outreach approaches and uptake of PCC

Intervention		Outreach	Uptake
Outreach approach	Number of municipalities that adopted the approach	Number reached by the approach	Number of PCC applications indicating this approach^a^
Municipal letters	7/10	110,199 letters	338
GP letters	10/10	21,930 letters	95
Child healthcare leaflets	8/10	unknown no. of leaflets	6
Peer health education	7/10	147 sessions; 1796 participants	1

Uptake was registered between February 2013 and the end of December 2014, following the implementation of a outreach approach per municipality

^a^Does not count up to the total number of 587 PCC applications due to missing data, overlap and other reported approaches

#### The effect of the outreach strategy

The total registered uptake following the outreach strategy consisted of 587 applications for a PCC consultation. This number differs from the sum of the uptake numbers reported in Table 1 for the following reasons: The outreach approach was not reported in 54 (9.2%) of the cases; nine women (1.5%) were reached by more than one of the four predefined outreach approaches; 102 women (17.4%) reported that another motivating factor than the four components of the outreach strategy had brought them to make the appointment. These women reported that they had made an appointment after being informed about PCC consultations by their midwife or their GP (other than by means of the letter), by friends or by different media (e.g. newspaper articles or websites).

When the uptake numbers are related to the outreach of all approaches, the effect is small. The relatively small-scale outreach activity of the child healthcare services and peer health educators resulted in hardly any applications (*n* = 7) for PCC. The mailings of letters informing women of PCC were the most effective measures since they resulted combined in 424 (72%) of the total applications for PCC. When we relate the uptake of the municipal letters (338) to the average annual number of pregnancies in the targeted areas of these municipalities (6875), the equivalent of 4.9% of these pregnant women would have been reached by PCC as a result of the letters.

Additional file 4 shows the timing of the municipal letter mailings in relation to the subsequent PCC appointments that were a result of these letters during the following year. Visualization shows that the main effect was seen in the first 3 months after the letter was sent and then seems to fade out.

### Characteristics of the women who utilized the PCC services

**Fig. 2:**
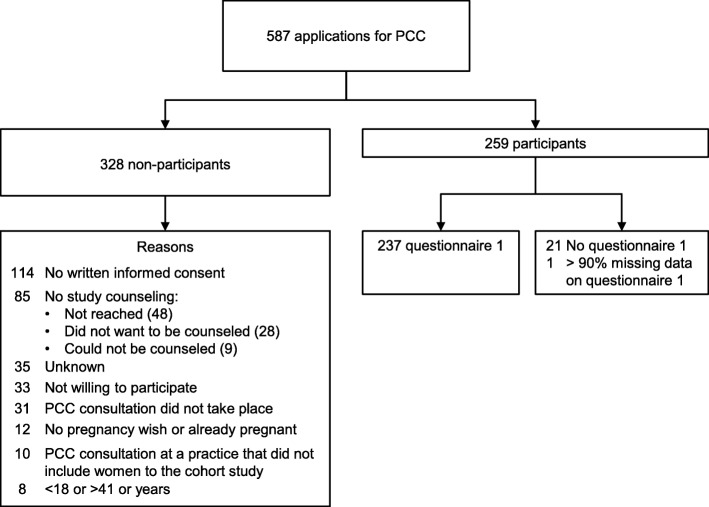
Participant enrollment in the cohort study

The enrollment and data collection process of the HP4All cohort study is presented in Fig. 2. Of the total of 587 women who applied for a PCC consultation, 259 women (44%) could be included in the cohort study. Reasons for exclusion or non-participation are described in Fig. 2. An important factor for exclusion was lack of written informed consent (*n* = 114). Of the 259 participants, 237 (92%) filled in questionnaire 1. Their characteristics are presented in Table 2 (and more detailed regarding their attitude and knowledge in Additional file 5).

**Table 2:** “Predisposing, enabling and need” characteristics of participants of the cohort

Socio-demographic characteristics (*N* = 237)^a^			Number*	(%)*
Age	Median age in years	(min–max)	30	(19–41)
		(IQR)		(27–34)
Ethnicity^b^	Dutch		145	(63.3)
Civil status	Married or living together		178	(77.1)
	In a relationship, not living together		32	(13.8)
	Not in a relationship		21	(9.1)
Educational attainment^c^	Low		18	(7.8)
	Intermediate		84	(36.5)
	High		121	(52.6)
	Other – foreign education		7	(3.1)
Occupational status	No paid job		53	(22.8)
Monthly household income (*N* = 212)	Low (< 1500€)		46	(21.7)
	Middle (1500–2500€)		65	(30.7)
	High (> 2500€)		101	(47.6)
Attitude and knowledge about PCC				
Barriers summary^d^ (max 25)	Median score (IQR)		12	(11–14)
Beliefs summary^e^ (max 45)	Median score (IQR)		37	(35–45)
Knowledge summary^f^ (max 8)	Median score (IQR)		6	(5–7)
Pregnancy and preconception health characteristics				
Pregnancy intention	Currently pregnant		4	(1.8)
	Within next 3 months		114	(50.4)
	Within next 3–6 months		59	(26.1)
	After > 6 months or maybe no intention		49	(21.7)
Subfertility	Current or previous fertility treatment		21	(9.0)
Previous pregnancy	Yes		69	(29.2)
Adverse pregnancy outcomes^g^	Miscarriage		23	(33.3)
	Abortion		22	(31.9)
	Low birth weight baby (< 2500 g)		7	(10.1)
	Child with congenital abnormalities		3	(4.3)
	Preterm birth (< 37 weeks)		4	(5.8)
	Perinatal mortality		1	(1.5)
Preconception lifestyle risks	No folic acid supplementation		83	(35.6)
	Smoking		30	(12.9)
	Alcohol consumption ≥1/week		51	(22.2)
	Illicit drug use		6	(2.6)
	No daily vegetables or fruit consumption		66	(28.4)
Self-rated health^h^	Moderate – poor		24	(10.3)

*Unless stated otherwise

^a.^In case of > 5% missing on an item, the number of participants that responded to the question is provided

^b.^Self-defined ethnicity

^c.^Educational attainment level was defined as the highest completed educational level classified according to the International Standard Classification of Education (ISCED) i.e. low (level 0–2: early childhood; primary education; lower secondary education); intermediate (level 3–5: upper secondary; post-secondary; short cycle tertiary); and high (level 6–8: bachelor; master; doctoral). Unesco institute for statistics 2014

^d.^Median sum score of five questions on attitude and potential barriers for uptake of PCC (minimum 5 – maximum 25). High score indicates high level of potential barriers. *N* = 214

^e.^Median sum score of nine questions on beliefs regarding PCC (minimum 9 – maximum 45). High score indicates positive attitude. *N* = 215

^f.^Median sum score of eight questions on knowledge of PCC risk factors (minimum 0 – maximum 8). High score indicates good knowledge. *N* = 220

^g.^Adverse pregnancy outcomes are presented as women who have experienced ≥1 time(s) specified outcomes

^h^Self-rated health was questioned as: How would you in general rate your health? (excellent-very good-good-moderate-poor)

#### Socio-demographic characteristics

Those who made use of PCC included women from nearly the total age range of the predefined target population. More than a third of women considered themselves from ethnic minorities, the largest proportion being from Surinamese background. Not only women in a relationship, but also single women made use of PCC. With regard to socio-economic status (SES) based on education, income and occupational status, the majority of the group consisted of women of higher SES, but women with lower SES characteristics also made use of a PCC consultation.

#### Barriers, beliefs and knowledge with regards to preconception health and PCC

With regards to attitudes towards a PCC consultation, the women in the cohort generally scored low on potential barriers to using PCC. However, two-thirds of the participants indicated that they would search for information about having a healthy pregnancy in alternative ways to the PCC consultation and one-third indicated they had enough knowledge already. The majority of women had positive beliefs and attitudes towards PCC. More than 84% of the women knew the right answer (true or false) to the knowledge statements on folic acid supplementation, medication and illicit drug use in relation to (early) pregnancy. By contrast, only half of the women knew the negative effects of smoking and being underweight on the success of conception.

#### Need and motivations for utilizing PCC services

**Fig. 3:**
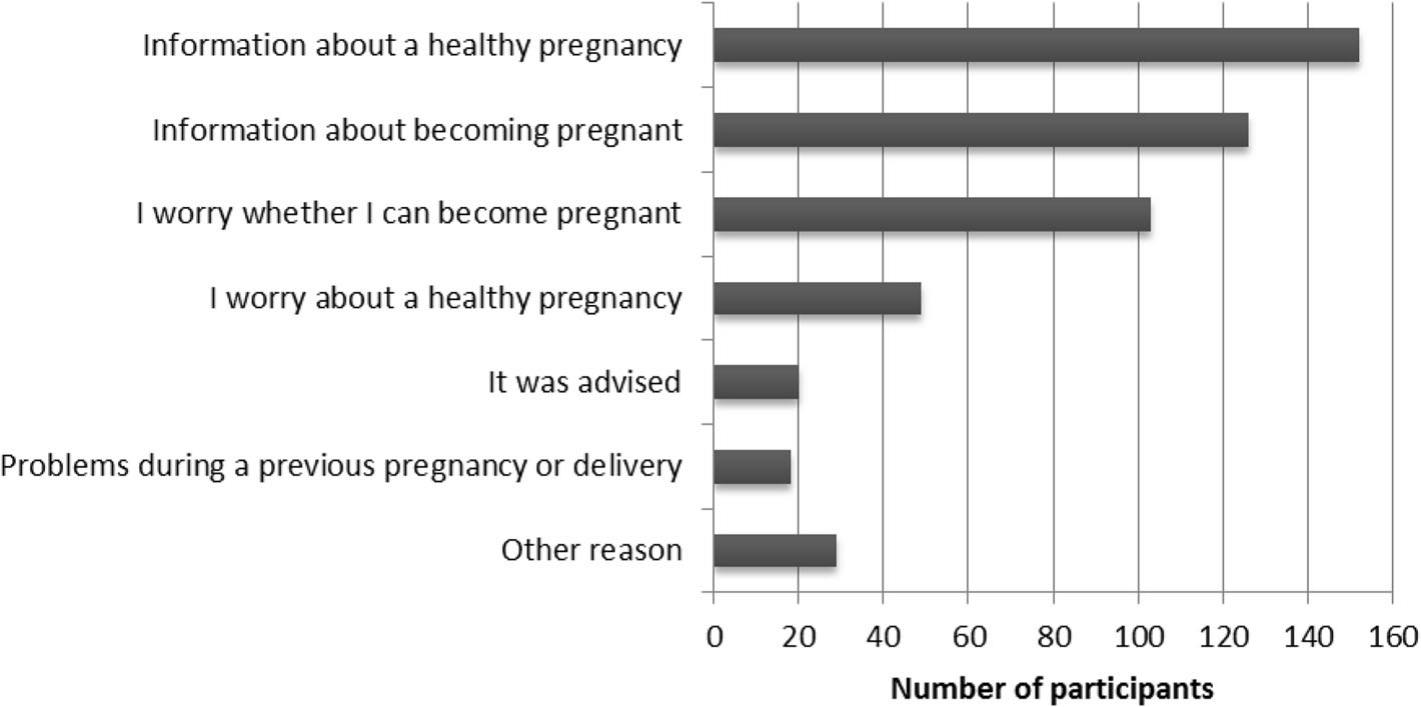
Reasons to apply for a PCC consultation. Participants could choose multiple reasons; three participants did not give any reason (*n* = 234)

Considering the need for PCC, we found that about half of the participants were planning to get pregnant within the next 3 months and about 10 % had fertility problems. Within the group who had been pregnant before (*n* = 69; 29%), considerably high percentages had experienced adverse pregnancy outcomes. In terms of behavioral risk levels, 82.3% had at least one of the five preconception lifestyle risk factors. To get an indication of women’s perceived need and motivation for uptake of PCC, we looked at which of the predefined reasons to utilize PCC applied (Fig. 3). Reasons relating to information and concerns about a healthy pregnancy and fertility were mentioned most. Additionally, women mentioned other reasons for utilizing PCC that included “because it was offered” and very specific questions regarding health issues or oocyte preservation.

## Discussion

### Principal findings

Our study illustrates how challenging it is to recruit women in the general population for PCC consultations in primary care. We measured the effect of the four-pronged outreach strategy in different ways. Firstly, regarding the uptake, the outreach resulted in a considerable number of applications for PCC (*n* = 587). To date, this is the largest preconception cohort recruited in primary care in the Netherlands. Most of the applications were a result of the large-scale mailing of letters targeting all women between 18 and 41 years. In relation to the reach of the outreach strategy, the effect seems small, but this is to be expected since the majority of these women would not actually consider becoming pregnant within the course of the study. We also found that the effect was mainly seen during a brief period of time following the mailing. Lastly, regarding the characteristics of women who applied for PCC, the strategy seems to have affected a diverse group of women. We reached a general population that aimed to conceive, as well as a subgroup of women with prior adverse pregnancy outcomes. Although more women with a higher educational attainment were recruited, the outreach strategy led to women with different socioeconomic backgrounds and different motivations applying for a PCC consultation.

### Comparison to previous findings

Prior to the study, uptake of PCC consultations offered by GPs and midwives was low [9]. In the absence of other outreach strategies, the consultations registered in our study can be attributed predominantly to the intervention. In other words, our outreach intervention resulted in a considerable increase of PCC delivery. The need for proactive outreach in order to educate about PCC services has also been illustrated by the low awareness regarding preconception health and PCC that has been found in previous studies [12, 22–24]. Combining PCC outreach or recruitment strategies, such as in our intervention, has been suggested before to improve delivery of PCC both in daily practice as well as in PCC studies [10, 25].

To our knowledge, a combination of the four outreach approaches in our strategy has not been evaluated before. However, some of the approaches have been implemented similarly before. Previous implementation of mailings about PCC from municipalities and GPs has also demonstrated a positive effect on uptake of PCC [18, 19]. One of these studies is in outline comparable to our approach of sending letters by GPs, but led to about 2.2% of the invited women attending PCC in contrast to 0.4% in our study [19]. Possibly, women in our study underreported this approach due to overlap with the municipal letters. Other studies have also recommended our other two approaches of integrating PCC in child healthcare and peer education before [20, 21, 25–27]. Regarding the effect of the different outreach or recruitment approaches, Velott, Baker, Hillemeier, Weisman [25] have provided an overview of previous studies involving various types of health promotion. They indicate that there is not a single “best” method, but differentiate between active (or personal), and passive methods. Passive approaches such as mass mailings have the advantage of recruiting larger numbers of participants in absolute terms, as seen in our study as well. However, active approaches have the advantage of being able to give further information to the target population [25]. In our study, active approaches such as peer education hardly resulted in any PCC applications, but might in itself already have fulfilled part of the purpose of PCC by educating women about preconception health.

Besides the predefined components of our outreach strategy, about 17% of the women in our study reported that other factors triggered them to apply for PCC. The most mentioned factor was information from their GP or midwife. This could indicate that raised awareness of healthcare professionals improves uptake of PCC. Furthermore, this is in line with prior findings that women like to be informed about PCC by a (primary) healthcare professional [24, 28, 29]. Opportunistic outreach by healthcare professionals during routine visits of clients may be complementary to the studied outreach strategy and valuable in reaching individuals with known risk factors, but on its own it does not guarantee reaching everyone.

In literature, it is often mentioned that reaching women who do not perceive a need for PCC (despite their risks) and who do not prepare for pregnancy is challenging [12, 30]. Our outreach intervention entailed a general approach since PCC is considered relevant for all women who consider getting pregnant [17]. We applied Andersen’s model of healthcare utilization to reflect upon factors that likely influence application for PCC (see Additional file 1). This shows that the PCC services mainly reached women with good preconception health knowledge and a positive attitude towards PCC. Two main reasons for utilizing PCC were optimizing chances for a healthy pregnancy and fertility concerns. It has been proposed to integrate fertility concerns into PCC to meet the needs of women [28]. With respect to the objective need for PCC, our cohort included women with social, obstetric or behavioral risk factors.

### Study strengths and limitations

Applying different outreach approaches for PCC simultaneously was a key attribute of the study and has not been performed at this scale in the Netherlands before. The four-pronged strategy was implemented and evaluated in a real-time setting of different municipalities. This provided the opportunity to create awareness on the importance of perinatal health and promote PCC in these communities via existing stakeholders across medical and social domains [31].

At the same time, this design brought about challenges as well. Context factors (e.g. local policies) led to variation in the implementation of the outreach strategy across municipalities. For instance, not all municipalities and GP practices sent letters, and the targeted population included some women outside the designated areas and age range (e.g. peer education sessions could be integrated in other meetings where older women were present as well). Adapting the intended intervention to suit local settings reduces fidelity and completeness of the implementation [32]. Understanding these mechanisms is important when evaluating effectiveness and qualitative analyses will be pursued to further explore the effect of the intervention.

There were a few limitations in the analysis of PCC uptake. We relied on participating practices to register appointments and respective outreach approaches, which was susceptible to unreliable registration. We did not have information about possible PCC consultations at non-participating practices and the outreach approach was not reported in 9 % of the appointments. In addition, we measured uptake for a brief, limited and varying period in each municipality. We believe we captured most of the effect, as we demonstrated that the effect faded out within the study period. Nevertheless, we only captured the effect of the outreach strategy in terms of uptake of PCC consultations and were not able to measure possible direct effects in terms of improved awareness or lifestyle changes regarding behavioral risks. For instance, the outreach approaches might have triggered women to look for more information without applying for a PCC consultation.

To reflect upon the population that utilized the PCC services, we relied on the cohort study [17]. However, the participation rate in this cohort study was low (44%). Consequently, data might have been susceptible to selection bias. Data considering behavioral risk factors could have been influenced by the timing of filling in the questionnaire in relation to the actual PCC consultation. Half of the participants filled in the questionnaires after the consultation. This would most likely have resulted in underreporting of behavioral risks. Ideally, this study would have been able to compare characteristics of women who applied for PCC after outreach compared to characteristics of women who did not respond to the outreach. However, as the mailing was sent to all women 18–41 years, the Medical Ethical Committee deemed a non-response study too intrusive and inappropriate.

## Conclusion

Based on this large community based intervention studied in ‘high risk’ municipalities, we conclude that an extensive four-pronged outreach strategy amongst the general population promotes uptake of PCC. However, this effect seems temporary and small. Efforts need to be continued to maintain and enlarge the uptake of PCC. To increase uptake, repetition or the continuous application of simultaneous outreach strategies is needed [18, 19]. The effectiveness of outreach strategies needs to be evaluated in light of implementation data to fine-tune the strategies. Tailoring outreach strategies to the needs of the population could potentially increase effectiveness and ensure subgroups specifically at risk of adverse pregnancy outcomes are reached.

Our outreach strategy has likely increased awareness of PCC to a larger extend than the measured PCC consultations. In future interventions, uptake and effectiveness of different PCC forms could be considered as well, for instance participation in PCC group education sessions, consulting websites, or using integrated PCC through online or mHealth platforms [33, 34]. Promoting PCC in various forms, in various ways and at various times will likely contribute to PCC uptake and ultimately to improved preconception health.

## Additional files

Additional file 1: The Framework of the Healthy Pregnancy 4 All PCC study. (PDF 141 kb)

Additional file 2: Example of a municipal PCC invitational Letter. (PDF 159 kb)

Additional file 3: English language version of the participant questionnaire. (PDF 765 kb)

Additional file 4: Figure showing the uptake of PCC applications after sending municipal invitation letters over time. (PDF 423 kb)

Additional file 5: Barrier, beliefs and knowledge response per statement (*N* = 237). (PDF 352 kb)
