# Supplementary material for: The effect of a preconception care outreach strategy: the Healthy Pregnancy 4 All study
Source: BMC Health Serv Res. 2019 Jan 23;19:60. doi: 10.1186/s12913-019-3882-y (PMC6343258; doi:10.1186/s12913-019-3882-y)
Supplement: Supplementary file 1 — The Framework of the Healthy Pregnancy 4 All PCC study. (PDF 141 kb) [file 12913_2019_3882_MOESM1_ESM.pdf]

Additional file 1. The Framework of the Healthy Pregnancy 4 All PCC study [1]

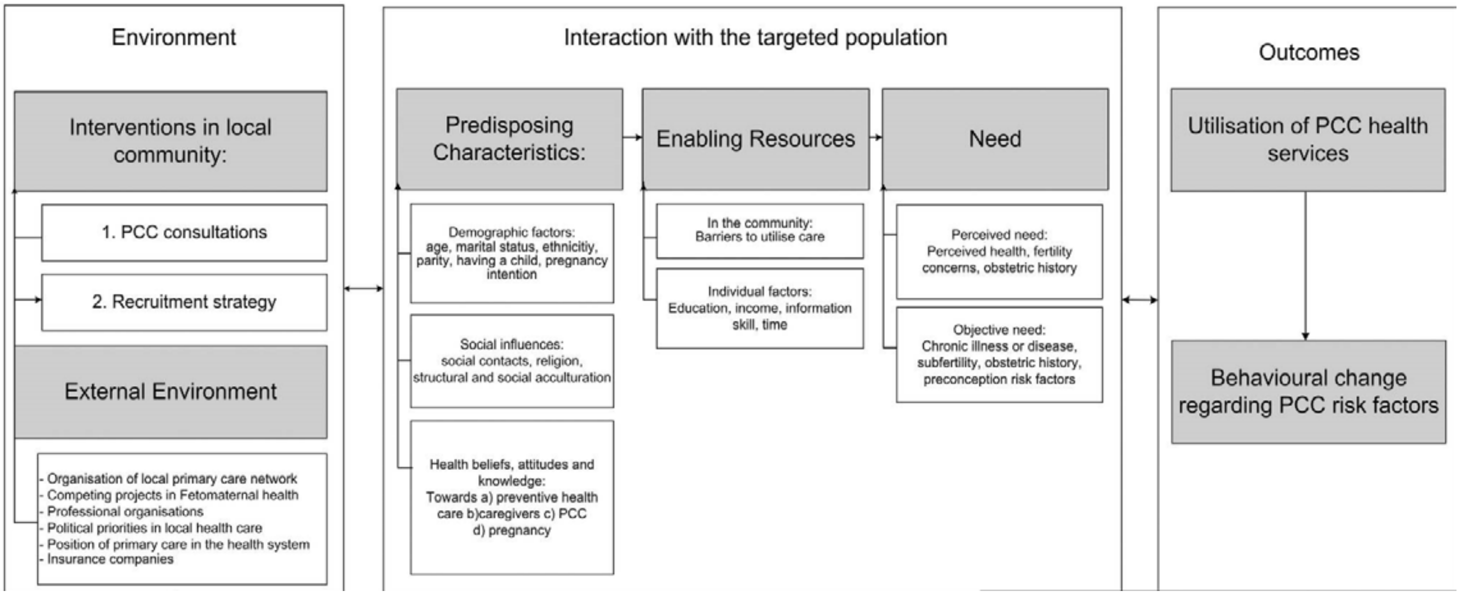


1. van Voorst SF, Vos AA, de Jong-Potjer LC, et al. Effectiveness of general preconception care accompanied by a recruitment approach: protocol of a community-based cohort study (the Healthy Pregnancy 4 All study). BMJ Open 2015;**5**(3):e006284.
