# Supplementary material for: The effect of a preconception care outreach strategy: the Healthy Pregnancy 4 All study
Source: BMC Health Serv Res. 2019 Jan 23;19:60. doi: 10.1186/s12913-019-3882-y (PMC6343258; doi:10.1186/s12913-019-3882-y)
Supplement: Supplementary file 2 — Example of a municipal PCC invitational Letter. (PDF 159 kb) [file 12913_2019_3882_MOESM2_ESM.pdf]

Additional file 2. Example of a municipal PCC invitational Letter

<u>Preconception care consultations</u> *Local LOGO*

<u>[literal translation from Dutch: child wish consultations]</u>

Dear Madam,

This letter is to inform you about preconception care consultations in [area]. These consultations are for women who think about having children. All women between the ages of 18 and 42 in your neighborhood receive this letter.

Perhaps becoming pregnant is difficult for you. Maybe you do not have an intention to become pregnant. If this letter is inappropriate or painful for you, we apologize. It is definitely not our intention to be hurtful.

Wat is a preconception care consultation?

The preconception care consultations are intended to guide and support women towards a healthy pregnancy. Women (or couples) with a pregnancy desire are welcome for a consultation and can expect:

- Personal advice before you become pregnant;
- Answers to questions about your health, fertility and becoming pregnant;
- Reduce health risks to your baby;
- A good preparation for pregnancy;

Do you want to make an appointment?
Are you considering to become pregnant? Make an appointment with one of the midwives or your GP when they offer preconception care (see the attached list with practices).

Research
If you make an appointment for a preconception care consultation, you will probably be asked whether you want to participate in the study 'Healthy Pregnancy 4 All', in other words: a healthy pregnancy for everyone! Participation in this study is not required.

More information?
For more information or if you have questions we refer you to … [website and/or local details]

Yours sincerely,
… [local municipal health director and Healthy Pregnancy 4 All program director]

In deze brief staat naar welke huisarts of verloskundige u kunt gaan.
Zij geven informatie en advies over zwanger worden en over een gezonde zwangerschap.

Give your child a healthy start in life and make an appointment for preconception care.
In this letter there is a list with general physicians and midwifes that offer preconception care.
They can advise you on getting pregnant and healthy pregnancy.

Gönnen Sie ihrem Kind einen guten Start und kommen Sie zu der Kinderwunschsprechstunde. In diesem Brief steht zu welchem Hausarzt oder zu welcher Hebamme Sie gehen können. Dort bekommen Sie Information und Beratung über wie Sie schwanger werden können und auch über eine gesunde Schwangerschaft.

Donnez un bon départ à votre enfant: venez à la consultation préconceptionnelle!
Dans cette lettre vous trouverez les noms des medecins de famille ou des sages-femmes aux quels vous pouvez vous adresser pour les soins préconceptionnaux. Ils vous renseigneront et vous conseilleront sur la manière dont vous pouvez tomber enceinte ainsique sur une grossese saine.

اعطو طفلكم بداية سليمة وتعالو إلى ساعة الاستقبال حول الرغبة في الإنجاب!

تجدون في هذه الرسالة ما هو طبيب المنزل أو القابلة يمكن التوجه إليه أو إليها.

إنهم سيقومون بالنصح و إعطاء المعلومات حول الإنجاب و سلامة الحمل.

Çocuğunuzun sağlığı için doğru bir başlangıç yapın ve hamile kalmak istiyorsanız görüş saatine gelin. Bu yazıda hangi aile hekimine veya ebeye gidebileceğiniz yazmaktadır. Onlar size hamile kalma ve sağlıklı hamilelik konularında bilgiler ve tavsiyeler verirler.

¡Bríndale un comienzo sano a tu bebe y acude a la consulta preconcepcional!
Encontrarás aquí el nombre del médico o de la matrona a quienes te puedes dirigir.
Te proporcionarán información y consejos para quedar embarazada y disfrutar de un embarazo sano.

Zapewnij Twojemu dziecku zdrowy start i odwiedź przychodnię dla osób planujących posiadanie dziecka!
Niniejsza korespondencja zawiera informacje o lekarzach rodzinnych oraz położnych, do których można się udać.
Udzielają oni informacji oraz poradnictwa na temat zajścia w ciążę oraz prawidłowego jej przebiegu.

Ако желаете детето ви да има здравословен старт, посетете приемния час по въпроси, свързани с желанието да забременеете! [kinderwensspreekuur]
В това писмо ще намерите списък със семейни лекари и акушерки, които бихте могли да посетите.
Те предлагат информация и съвети за забременяване и здравословна бременност.
