# Supplementary material for: The effect of a preconception care outreach strategy: the Healthy Pregnancy 4 All study
Source: BMC Health Serv Res. 2019 Jan 23;19:60. doi: 10.1186/s12913-019-3882-y (PMC6343258; doi:10.1186/s12913-019-3882-y)
Supplement: Supplementary file 3 — English language version of the participant questionnaire. (PDF 765 kb) [file 12913_2019_3882_MOESM3_ESM.pdf]

Questionnaire 1

Registration information:	
1. What is your date of birth?	☐☐ - ☐☐ - ☐☐☐☐ [DD-MM-YYYY]
2. What date is it today?	☐☐ - ☐☐ - ☐☐☐☐ [DD-MM-YYYY]
3. What is your postal code?	☐☐☐☐

PART 1: Your visit to the Pre-Pregnancy Clinic:

The following questions are about your visit to the Pre-Pregnancy Clinic.

1. What was the most important reason for you to visit the Pre-Pregnancy Clinic? You can choose multiple answers.

- □ I would like information about becoming pregnant
- □ I would like information about a healthy pregnancy
- □ I worry whether I can become pregnant
- □ I worry about a healthy pregnancy
- □ There were problems during my previous pregnancy
- □ There were problems during a previous delivery
- □ Because it was advised
- □ Other reason: ____________

2. How did you know about the Pre-Pregnancy Clinic? You can choose multiple answers.

- □ a letter from the Municipal Health Service
- □ a letter from my general practitioner
- □ the preventive child healthcare services
- □ my general practitioner
- □ my midwife
- □ another healthcare provider
- □ a peer health educators
- □ my partner
- □ a family member
- □ a friend
- □ someone from my neighborhood
- □ someone from my church, synagogue, mosque or temple
- □ a poster in the GP practice
- □ a poster in midwifery practice
- □ a leaflet in de GP practice
- □ a leaflet in midwifery practice □ de krant
- □ a magazine
- □ a webpage
- □ www.zwangerwijzer.nl
- □ other: ____________

3. Do you know what a Peer (perinatal) Health Educator is?

- □ no (proceed to question 5)
- □ yes

4. What did the Peer Health Educator do for you?

- □ I did not use the Peer Health Educator
- □ I had a personal conversation with the Peer Health Educator
- □ I attended a group information meeting
- □ The Peer Health Educator made an appointment for me at the Pre-pregnancy Clinic
- □ The Peer Health Educator filled out www.zwangerwijzer.nl with me
- □ The Peer Health Educator joins me on my visit to the midwife

5. Explanation: A Peer (perinatal) Health Educator provides information about becoming pregnant and a healthy pregnancy. The peer educator can also help you to make an appointment at the Pre-Pregnancy Clinic. And she can go with you to the doctor or midwife.

If you had known what a Peer Health Educator could do for you, would you have used it?

□ no □ yes

6. Have you already discussed your desire to become pregnant with another health care provider? You can choose multiple answers.

□ No □ with my GP □ with a midwife □ with a gynecologist □ other:

7. How much faith do you have in health care providers when it comes to discussing your desire to become pregnant?

Your GP	A midwife	A gynecologist:
□ very much	□ very much	□ very much
□ much	□ much	□ much
□ not much/ not little	□ not much/ not little	□ not much/ not little
□ little	□ little	□ little
□ very little	□ very little	□ very little

8. Have you consulted other about becoming pregnant and a healthy pregnancy?
You can choose multiple answers:

□ no □ yes, family □ yes, friends

9. If I had to, I would pay for the Pre-Pregnancy Clinic:

□ no □ yes, I would pay up to EURO

PART 2: Your health

The following questions are about your health, your desire to become pregnant, your fertility and possible previous pregnancies.

1. In general would you say your health is:<br>□ excellent □ very good □ good □ fair □ poor
2. During a typical day, are you limited in activities of moderate effort, such as moving a table, pushing a vacuum cleaner or swimming?<br>□ yes, limited a lot □ yes, limited a little □ no, not limited at all
3. During a typical day, are you limited in climbing several flights of stairs?<br>□ yes, limited a lot □ yes, limited a little □ no, not limited at all
4. Have you accomplished less in the past 4 weeks than you would like as a result of your physical health?<br>□ no □ yes
5. Have you been limited in your work or other common activities as a result of your physical health during the past 4 weeks?<br>□ no □ yes
6. Have you been able to do or achieve less in the past 4 weeks than you would like as a result of emotional problems (such as feeling depressed or anxious)?<br>□ no □ yes
7. Did you not deal with your work or other activities as carefully as usual due to emotional problems?<br>□ no □ yes
8. During the past 4 weeks, how much did pain interfere with your normal daily activities?<br>□ not at all □ a little bit □ moderately □ Quite a bit □ Extremely
9. How much of the time during the past 4 weeks have you felt calm and peaceful?<br>□ not at all □ a little bit □ moderately □ Quite a bit □ Extremely
10. How much of the time during the past 4 weeks did you have a lot of energy?<br>□ not at all □ a little bit □ moderately □ Quite a bit □ Extremely
11. How much of the time during the past 4 weeks have you felt downhearted and blue?<br>□ not at all □ a little bit □ moderately □ Quite a bit □ Extremely
12. During the past 4 weeks, how much of the time has your physical health or emotional problems interfered with your social activities (like visiting with friends, relatives)<br>□ not at all □ a little bit □ moderately □ Quite a bit □ Extremely

13. Has a doctor ever diagnosed one of these conditions with you?<br>You can choose multiple answers.<br>□ arteriosclerosis coronary arteries / chest pain<br>□ heart attack<br>□ heart failure<br>□ cerebral infarction or stroke<br>□ arteriosclerosis in your legs<br>□ a congenital heart defect<br>□ no, none of the above
14. Are you currently using medication to lower your blood pressure?<br>□ no □ yes
15. Have you ever measured too high blood sugar?<br>For example, during a disease, a medical examination or a previous pregnancy<br>□ no □ yes
16. Do you have diabetes (diabetes type 1 or 2)?<br>□ no □ yes
17. Does someone in your family have diabetes (type 1 or 2)?<br>□ no □ yes, one or both parents, brother, sister or own child<br>□ yes, other family members grandmother, aunt, uncle, nephew / niece
18. Was there any of the following conditions in one of your pregnancies?<br>You can check multiple answers:<br>□ not applicable: I have not been pregnant before<br>□ high blood pressure or pre-eclampsia<br>□ gestational diabetes<br>□ no, none of the above

PART 3: Your Lifestyle

This part of the questionnaire is about your lifestyle.

1. How often do you eat vegetables or fruit?<br>□ every day<br>□ not every day
2. How much fruit do you usually eat per day?<br>☐ Units<br>Explanation: 1 unit of fruit is:<br>1 larger piece of fruit such as an orange, apple, pear, banana, grapefruit<br>2 smaller pieces of fruit: such as plums, apricots, kiwis<br>6-8 small pieces of fruit such as: strawberries, blackberries, raspberries, grapes
3. How much servings of vegetables do you usually eat a day?<br>☐ servings
4. Do you usually eat 100 grams of fish more than twice a week?<br>□ no □ yes
5. Do you usually eat more than 90 grams of whole grain products a day?<br>□ no □ yes<br>Explanation: What is 90 grams?<br>1 whole meal sandwich or rye bread = 30 grams<br>1 serving of muesli = 40 grams<br>1 portion of whole-wheat paste = 100 grams
6. You drink more than 450 kilocalories (Kcal) per week in drinks with added sugars<br>□ no □ yes<br>Note: If you drink more than 8 glasses of lemonade or 3 cans of soft drinks (not light) per week, you can enter yes. (1 can (350 ml) soft drink (not light) = 150 Kcal, 1 glass of lemonade (150 ml) = 60 Kcal)
7. Do you follow a strict low-salt diet? □ no □ yes

8. Are you taking folic acid tablets at the moment?<br>□ no, *continue to question 11*<br>□ yes, tablets of 0.4 or 0.5mg folic acid.<br>□ yes, tablets of 4 or 5mg folic acid (special high dosage)

9. Since how long do you take folic acid tablets?

☐ weeks

10. How often do you take folic acid tablets?

□ every day

□ 1 to 3 times a week

□ 4 to 6 times a week

□ less than once a week

11. Do you use multivitamin tablets?

□ no: go to question 14

□ yes, multivitamin for pregnant women

□ yes, multivitamin not specifically for pregnant women

12. Since how long do you take multivitamin tablets?

☐ weeks

13. How often do you take multivitamin?

□ every day

□ 1 to 3 times a week

□ 4 to 6 times a week

□ less than once a week

---

14. Do you use vitamin D tablets?

□ no

□ yes, how much vitamin D is there in 1 tablet? □ ☐ IU (international units)

□ ☐ microgram/ µg

15. Do you use cod liver oil or fish oil tablets?

□ no □ yes

16. Do you have any of these bowel diseases? You can tick multiple answers.

□ Ulcerative colitis

□ Crohn's disease

□ Celiac disease or gluten allergy

□ a spastic intestine

□ no, none of the above intestinal diseases

17. Do you wear a headscarf or veil?

□ no □ yes

18. Do you move for at least 30 minutes per day (in your daily activities or during exercise)?

□ no □ yes

20. How much exercise do you get per week?

☐ minutes of moderate physical exercise
(Moderate exercise is for example: walking, cycling, housework, quiet swimming)

☐ minutes of heavy physical exercise
(For example, heavy exercise is: running, racing bikes, swimming competitions, jumping rope, heavy work in the garden or at home)

21. Do you smoke?

□ no, I have never been a smoker: go to question 27

□ no, I am an ex-smoker: go to question 22

□ yes, occasionally: go to question 23

□ yes, daily: go to question 23

22. Since when did you quit?

Since ☐☐ - ☐☐ - ☐☐☐☐ [DD-MM-YYYY]; go to question 24

23. Are you trying to quit now?

□ yes, I am trying to quit since: ☐☐ - ☐☐ - ☐☐☐☐ [DD-MM-YYYY]

□ I reduce my smoking: go to question 25

□ no, I smoke as always: go to question 26

24. Have you smoked since your attempt to quit (even if it was only 1 cigarette or roll)?

□ no □ yes

25. Do you use anything to stop or reduce smoking?

□ no □ yes:

□ with a non-smoking course or group therapy

□ a smoking cessation clinic

□ a special stop smoking program from the midwife

□ I use nicotine chewing gum

□ I use nicotine patches

□ I use nicotine nasal spray

□ I take pills / a medication

□ other: ☐

26. How much do you smoke on a normal day? ☐ cigarettes / shags / cigars / cigarillos per day

27. Does your partner smoke?<br><br>□ No, he has never been a smoker<br>□ no, he is an ex-smoker<br>□ yes, occasionally<br>□ yes, daily
28. How long is there a total of smoking around you on a normal day? For example at home, at work or on occasions.<br><br>□ rarely / never<br>□ more than 3 hours a day<br>□ 1 to 3 hours per day<br>□ less than 1 hour per day

29. How often do you drink alcoholic beverages?<br><br>□ never, go to question 39<br>□ less than 1 glass per week<br>□ 1 to 3 glasses per week<br>□ 4 to 6 glasses per week<br>□ 1 glass per day<br>□ 1 to 3 glasses per day<br>□ more than 3 glasses per day
30. Have you ever drunk more than 6 glasses in the past 3 months?<br><br>□ no<br>□ yes, that has happened in □ times in the past 3 months
31. How many glasses of alcohol did you drink last week?<br>□□ Beer or wine<br>□□ Liquor
32. How often does your husband drink alcoholic drinks on a normal day on which he drinks?<br><br>□ never<br>□ less than 1 glass per week<br>□ 1 to 3 glasses per week<br>□ 4 to 6 glasses per week<br>□ 1 glass per day<br>□ 1 to 3 glasses per day<br>□ more than 3 glasses per day

33. Do you use drugs?<br>□ no, never: go to question 35<br>□ no, I stopped more than 1 week ago<br>□ no, I stopped less than 1 week ago<br>□ yes

34. Check what and how often you use (or used) drugs:

Marijuana<br>(Hash, weed, Dutch weed, marijuana, skunk, stuff)	□ daily<br>□ weekly<br>□ monthly	□ Amphetamines<br>*(Pep, Speed)*	□ daily<br>□ weekly<br>□ monthly	□ Phenycyclidine<br>*(Angel Dust)*	□ daily<br>□ weekly<br>□ monthly
□ Cocaine<br>*(coke)*	□ daily<br>□ weekly<br>□ monthly	□ Methamphetamine	□ daily<br>□ weekly<br>□ monthly	□ GHB	□ daily<br>□ weekly<br>□ monthly
□ Heroin	□ daily<br>□ weekly<br>□ monthly	□ Methadone<br>*(Symoron)*	□ daily<br>□ weekly<br>□ monthly	□ Ecstasy<br>*(XTC)*	□ daily<br>□ weekly<br>□ monthly
□ Another drug: namely:				□ daily<br>□ weekly<br>□ monthly	

35. Are you actively doing something now to gain weight?<br>□ neo<br>□ yes:<br>□ I visit a dietitian<br>□ I have changed my diet<br>□ something else: namely:
36. Are you actively doing something to lose weight now?<br>□ no<br>□ yes:<br>□ I visit a dietitian<br>□ I move or exercise more<br>□ I have changed my diet<br>□ I use a means to lose weight (for example: pills, drinks)<br>□ I visit a clinic<br>□ something else: namely:

37. Do you change the litter box at home?<br>□ no □ yes □ I do not have a cat or litter box
38. Do you garden?<br>□ no<br>□ yes, without gloves<br>□ yes, with gloves<br>□ I do not have a garden

39. When is it advisable to stop habits that can be harmful to the baby in the womb?

□ when stopping contraception

□ after unprotected sexual intercourse

□ after the pregnancy test is positive

□ do not know

PART 4: pregnancy planning and fertility

This part of the questionnaire is about planning a pregnancy and fertility.

PREGNANCY PLANNING

1. Do you or your partner use contraceptives?
   - □ no
   - □ yes: enter which contraceptives you use:
     - □ the birth control pill
     - □ IUD with hormone
     - □ IUD without hormones
     - □ condoms
     - □ contraceptive injections
     - □ the contraceptive implant (in the arm)
     - □ sterilization
     - □ other: ☐

2. Since when are you trying to become pregnant?
   - □ not yet
   - □ since we stopped using contraceptives: month: ____________year: ☐☐☐☐
   - □ other, since: month: ____________year: ☐☐☐☐

3. What are your thoughts on becoming pregnant?
   - □ I am pregnant
   - □ I intend to become pregnant:
     - □ within the next 3 months
     - □ within the next 3 – 6 months
     - □ after 6 months
   - □ maybe I decide not to become pregnant

FERTILITY

4. How long is your menstrual cycle?
   Explanation: This is the time between the start of the bleeding until the next bleeding.
   - □ shorter than 3 weeks
   - □ 3 to 5 weeks
   - □ longer than 5 weeks, but regularly
   - □ irregular
   - □ I do not know

5. Are you currently undergoing treatment to become pregnant?
   - □ no
   - □ yes

6. Have you previously had treatments to become pregnant?
   - □ no
   - □ yes

PART 5: Your pregnancies

This part of the questionnaire is about whether you have been pregnant.

PREGNANCY

1. Have you been pregnant before?

Explanation: a miscarriage or abortion also count as a pregnancy

□ no

continue to part 6

□ yes: *continue to question 2A/2B/2C/ 2D*

The following applies: If you have had twins (or triplets), you can enter this as two deliveries. By the date of birth of the babies, we know that they were twins.

2A Your first pregnancy:

2A1 Fill in when and how your pregnancy ended:

□ a full-term pregnancy with a delivery on	□□ - □□ - □□□□<br>DD – MM - YYYY
□ a miscarriage<br>*Continue to 2B*	month: ____________ year: □□□□
□ an ectopic pregnancy<br>*Continue to 2B*	month: ____________ year: □□□□
□ an abortion (at own request)<br>*Continue to 2B*	month: ____________ year: □□□□
□ a termination of pregnancy on medical grounds<br>*Continue to 2B*	month: ____________ year: □□□□

2 A2 Was your child: □ a boy □ a girl

2 A3 What was the weight of your baby at birth? □ gram

2 A4 How was your baby born?

- □ vaginal delivery (not assisted)
- □ assisted vaginal delivery
- □ caesarean section

Explanation: an assisted delivery is for example a vacuum-assisted or forceps-assisted delivery.

2 A5 Was your baby born prematurely?

- □ no, after the 37th week of pregnancy
- □ yes; the baby was born when I was … weeks pregnant

2 A6 Was there any of the following problems? You can choose multiple answers

- □ one or more congenital abnormalities
- □ your baby was stillborn when you were .... weeks pregnant
- □ your baby died in the first month after delivery

2 B Your second pregnancy:

2 B1 Fill in when and how your pregnancy ended:

□ a full-term pregnancy with a delivery on	□□ - □□ - □□□□<br>DD – MM - YYYY
□ a miscarriage<br>Continue to 2C	month: ____________ year: □□□□
□ an ectopic pregnancy<br>Continue to 2C	month: ____________ year: □□□□
□ an abortion (at own request)<br>Continue to 2C	month: ____________ year: □□□□
□ a termination of pregnancy on medical grounds<br>Continue to 2C	month: ____________ year: □□□□

2 B2 Was your child: □ a boy □ a girl

2 B3 What was the weight of your baby at birth? □ gram

2 B4 How was your baby born?

□ vaginal delivery (not assisted)

□ assisted vaginal delivery

□ caesarean section

Explanation: an assisted delivery is for example a vacuum-assisted or forceps-assisted delivery.

2 B5 Was your baby born prematurely?

□ no, after the 37th week of pregnancy

□ yes; the baby was born when I was … weeks pregnant

2 B6 Was there any of the following problems? You can choose multiple answers

□ one or more congenital abnormalities

□ your baby was stillborn when you were .... weeks pregnant

□ your baby died in the first month after delivery

2 C Your third pregnancy:

2 C1 Fill in when and how your pregnancy ended:

□ a full-term pregnancy with a delivery on	□□ - □□ - □□□□<br>DD – MM - YYYY
□ a miscarriage<br>Continue to 2D	month: ____________ year: □□□□
□ an ectopic pregnancy<br>Continue to 2D	month: ____________ year: □□□□
□ an abortion (at own request)<br>Continue to 2D	month: ____________ year: □□□□
□ a termination of pregnancy on medical grounds<br>Continue to 2D	month: ____________ year: □□□□

2 C2 Was your child: □ a boy □ a girl

2 C3 What was the weight of your baby at birth? □ gram

2 C4 How was your baby born?

□ vaginal delivery (not assisted)

□ assisted vaginal delivery

□ caesarean section

Explanation: an assisted delivery is for example a vacuum-assisted or forceps-assisted delivery.

2 C5 Was your baby born prematurely?

□ no, after the 37th week of pregnancy

□ yes; the baby was born when I was … weeks pregnant

2 C6 Was there any of the following problems? You can choose multiple answers

□ one or more congenital abnormalities
□ your baby was stillborn when you were .... weeks pregnant
□ your baby died in the first month after delivery

2 D Your fourth pregnancy:

2 D1 Fill in when and how your pregnancy ended:

□ a full-term pregnancy with a delivery on	□□ - □□ - □□□□<br>DD – MM - YYYY
□ a miscarriage<br>Continue to 3	month: ____________ year: □□□□
□ an ectopic pregnancy<br>Continue to 3	month: ____________ year: □□□□
□ an abortion (at own request)<br>Continue to 3	month: ____________ year: □□□□
□ a termination of pregnancy on medical grounds<br>Continue to 3	month: ____________ year: □□□□

2 D2 Was your child: □ a boy □ a girl

2 D3 What was the weight of your baby at birth? □ gram

2 D4 How was your baby born?

□ vaginal delivery (not assisted)

□ assisted vaginal delivery

□ caesarean section

Explanation: an assisted delivery is for example a vacuum-assisted or forceps-assisted delivery.

2 D5 Was your baby born prematurely?

□ no, after the 37th week of pregnancy

□ yes; the baby was born when I was … weeks pregnant

2 D6 Was there any of the following problems? You can choose multiple answers

□ one or more congenital abnormalities
□ your baby was stillborn when you were .... weeks pregnant
□ your baby died in the first month after delivery

3. How many living children do you have? □ children

PART 6: General information about you:

This part of the questionnaire is about you and your background.

1. Are you:	□ married<br>□ living together with partner	□ in a relationship but not living together<br>□ single

2. With whom do you share your household?

- □ with ☐ adults (count your partner)
- □ with ☐ children under the age of 18
- □ children under the age of 18

3. Do you have a paid job	□ no	□ yes: ☐ hours per week

4. What is the net income of your household per month in euros?

- □ less than 1000 euros per month
- □ 1000 to 1500 euros per month
- □ 1500 to 2000 euros per month
- □ 2000 to 2500 euros per month
- □ 2500 to 3000 euros per month
- □ more than 3000 euros per month

Explanation: Net income is: the amount that you receive on your account or in your hands. Calculate the income that comes in for all people in your household. In addition to your possible income, you can also include benefits or gifts through work.

5. Do you have health insurance?

- □ no
- □ yes

6. What is the highest level of education that you have completed?

- □ none
- □ primary school
- □ secondary education:
  - □ Preparatory vocational education (VBO)/preparatory vocational secondary education (VMBO) basic vocational or advanced vocational track
  - □ Junior general secondary education (MAVO)/preparatory vocational secondary education (VMBO) combined or theoretical track
  - □ Senior general secondary education (HAVO)
  - □ Pre-university education (VWO)
- □ Senior secondary vocational education (MBO)
- □ Higher professional education (HBO)
- □ University education (university/post HBO)
- □ Education abroad: ☐

7. Which population group do you consider yourself part of?

- □ Dutch
- □ Suriname-Creole
- □ Suriname-Hindustani
- □ Suriname-Javan
- □ Suriname - other: ______
- □ Antillean/Aruban
- □ Indonesian/Moluccan
- □ Turkish
- □ Kurdish
- □ Moroccan: Berbers
- □ Moroccan: Arabic
- □ Polish
- □ Other: ______

8. In which country were you born?

- □ The Netherlands
- □ Suriname
- □ Antilles/ Aruba
- □ Indonesia / the Moluccas
- □ Turkey
- □ Morocco
- □ Other: ______

9. In which country was your mother born?

- □ The Netherlands
- □ Suriname
- □ Antilles/ Aruba
- □ Indonesia / the Moluccas
- □ Turkey
- □ Morocco
- □ Other: ______

10. In which country was your father born?

- □ The Netherlands
- □ Suriname
- □ Antilles/ Aruba
- □ Indonesia / the Moluccas
- □ Turkey
- □ Morocco
- □ Other: ______

11. What is your mother tongue?

- □ Dutch
- □ Surinamese Sranan Tongo
- □ Surinamese Hindustani
- □ Papiamento
- □ Indonesian / Moluccan
- □ Turkish
- □ Kurdish
- □ Arabic
- □ Berbers
- □ Chinese, Mandarin
- □ English
- □ Other, namely: ______

12. How well can you understand Dutch?

□ not at all □ a little □ it goes □ reasonably good □ very well

13. How well can you read Dutch?

□ not at all □ a little □ it goes □ reasonably good □ very well

14. How well can you speak Dutch?

□ not at all □ a little □ it goes □ reasonably good □ very well

15. How well can you write Dutch?

□ not at all □ a little □ it goes □ reasonably good □ very well

16. Are you religious? □ n0 □ yes I am

□ Christian
□ Muslim
□ Hindu
□ Jewish
□ Buddhist
□ different ________

17. How often do you go to: a religious activity or organization?

□ every day □ 1x per week □ at least 1 x per month □ 1 or a few times a year □ less than 1 x per year □ not applicable

18. Do you have a partner? □ no *go to question: 23*

□ yes

19. In which year was your partner born? In : □□□□ (YYYY)

20. In which country was your partner born?

□ The Netherlands
□ Suriname
□ Antilles / Aruba
□ Indonesia / Moluccas
□ Turkey
□ Morocco
□ Other: ________

21. In which country was the mother of your partner born?

□ The Netherlands
□ Suriname
□ Antilles / Aruba
□ Indonesia / Moluccas
□ Turkey
□ Morocco
□ Other: ________

22. In which country was the father of your partner born?

□ The Netherlands
□ Suriname
□ Antilles / Aruba
□ Indonesia / Moluccas
□ Turkey
□ Morocco
□ Other: ________

23. How often do you have contact with family members?

□ every day □ 1x per week □ at least 1 x per month □ 1 or a few times a year □ less than 1 x per year □ not applicable

24. How often do you have contact with friends or acquaintances?

□ every day □ 1x per week □ at least 1 x per month □ 1 or a few times a year □ less than 1 x per year □ not applicable

25. How often do you have contact with Dutch friends or acquaintances?<br>□ every day □ 1x per week □ at least 1 x per month □ 1 or a few times a year □ less than 1 x per year □ not applicable
26. How often do you have contact with immigrant friends or acquaintances?<br>□ every day □ 1x per week □ at least 1 x per month □ 1 or a few times a year □ less than 1 x per year □ not applicable

PART 7: YOUR IDEAS ABOUT HEALTH, THE PRE-PREGNANCY CLINIC (PCC CONSULATION) AND PREGNANCY:

This part of the questionnaire is about your ideas and facts about health, getting pregnant and wanting to have children.

Statements are listed below. Tick the extent to which you agree or disagree with it.					
1. I take part or will participate in screening for cervical cancer after my 30th.	☐ Strongly agree	☐ Agree	☐ No opinion	☐ disagree	☐ Strongly disagree
2. I find it positive that you can visit a healthcare provider to discuss your pregnancy desire.	☐ Strongly agree	☐ Agree	☐ No opinion	☐ disagree	☐ Strongly disagree
3. I find it uneasy to discuss getting pregnant with my GP or midwife	☐ Strongly agree	☐ Agree	☐ No opinion	☐ disagree	☐ Strongly disagree
4. When you have a PCC consultation chances are greater that you will have a healthy pregnancy.	☐ Strongly agree	☐ Agree	☐ No opinion	☐ disagree	☐ Strongly disagree
5. It is not necessary to have a PCC consultation before you are pregnant	☐ Strongly agree	☐ Agree	☐ No opinion	☐ disagree	☐ Strongly disagree
6. Visiting a healthcare provider for a PCC consultation makes me medicalize 'becoming pregnant'	☐ Strongly agree	☐ Agree	☐ No opinion	☐ disagree	☐ Strongly disagree
7. Because of a PCC consultation I feel pressured to have a perfect baby	☐ Strongly agree	☐ Agree	☐ No opinion	☐ disagree	☐ Strongly disagree
8. I am afraid of having a PCC consultation because I am afraid of a gynecological examination	☐ Strongly agree	☐ Agree	☐ No opinion	☐ disagree	☐ Strongly disagree
9. I would have my child vaccinated in the national vaccination program.	☐ Strongly agree	☐ Agree	☐ No opinion	☐ disagree	☐ Strongly disagree
10. I do not appreciate that a healthcare provider interferes with my pregnancy desire	☐ Strongly agree	☐ Agree	☐ No opinion	☐ disagree	☐ Strongly disagree
11. I am afraid of negative responses from the people around me when I have a PCC consultation	☐ Strongly agree	☐ Agree	☐ No opinion	☐ disagree	☐ Strongly disagree
12. You owe it to your baby to do everything within your possibilities for the health of your baby.	☐ Strongly agree	☐ Agree	☐ No opinion	☐ disagree	☐ Strongly disagree
13. Because of my health and / or previous childbirth (s) there is a chance that my baby will be born prematurely.	☐ Strongly agree	☐ Agree	☐ No opinion	☐ disagree	☐ Strongly disagree
14. Because of my health and / or previous pregnancy (s) there is a chance that my baby has a congenital abnormality.	☐ Strongly agree	☐ Agree	☐ No opinion	☐ disagree	☐ Strongly disagree
15. Because of my health and / or previous childbirth (s) there is a chance that my baby does not grow well during pregnancy.	☐ Strongly agree	☐ Agree	☐ No opinion	☐ disagree	☐ Strongly disagree
16. It is difficult for me to visit a GP or midwife due to practical reasons (for example, not being able to take time off from work, not being able to find a babysitter, having no transport).	☐ Strongly agree	☐ Agree	☐ No opinion	☐ disagree	☐ Strongly disagree

17. I find it difficult to make an appointment with my GP or midwife at a suitable moment for me	□ Strongly agree	□ Agree	□ No opinion	□ disagree	□ Strongly disagree
18. It takes too much time for me to go to a preconception care consultation	□ Strongly agree	□ Agree	□ No opinion	□ disagree	□ Strongly disagree
19. I look for information to have a healthy pregnancy in other ways (e.g. internet)	□ Strongly agree	□ Agree	□ No opinion	□ disagree	□ Strongly disagree
20. I have enough knowledge about what to do to have a healthy pregnancy	□ Strongly agree	□ Agree	□ No opinion	□ disagree	□ Strongly disagree

**Statements are listed below. Tick the box that suits your opinion.**			
21. Whether you are healthy or not, everyone can improve something in preparation for a pregnancy.	□ True	□ Not true	□ I do not know
22. When you smoke, you become pregnant less quickly	□ True	□ Not true	□ I do not know
23. When you have a very low weight, you become pregnant less quickly	□ True	□ Not true	□ I do not know
24. When you have a very high weight, you become pregnant less quickly	□ True	□ Not true	□ I do not know
25. The Pre-pregnancy clinic (or a preconception care consultation) is intended for women who have difficulty getting pregnant.	□ True	□ Not true	□ I do not know
26. All medications that you can buy at a pharmacy are safe: you can take them during pregnancy	□ True	□ Not true	□ I do not know
27. You do not need to take folic acid supplementation until you know that you are pregnant	□ True	□ Not true	□ I do not know
28. The Pre-pregnancy clinic (or a preconception care consultation) is intended for women who have previously had a child who was not healthy.	□ True	□ Not true	□ I do not know
29. When you want to become pregnant, you should stop eating raw meat or fish	□ True	□ Not true	□ I do not know
30. When you want to become pregnant, you should stop eating a lot of liver	□ True	□ Not true	□ I do not know
31. The Pre-pregnancy clinic (or a preconception care consultation) is intended for women who have a disease themselves.	□ True	□ Not true	□ I do not know
32. Drugs are not yet harmful in the beginning of pregnancy	□ True	□ Not true	□ I do not know

PART 8: CLOSURE AND SPACE FOR COMMENTS

Thank you for completing the questionnaire for the Healthy Pregnancy 4 All study.

Do you have any comments about the questionnaire? You can write them down here.
