# Supplementary material for: The effect of a preconception care outreach strategy: the Healthy Pregnancy 4 All study
Source: BMC Health Serv Res. 2019 Jan 23;19:60. doi: 10.1186/s12913-019-3882-y (PMC6343258; doi:10.1186/s12913-019-3882-y)
Supplement: Supplementary file 4 — Figure showing the uptake of PCC applications after sending municipal invitation letters over time. (PDF 423 kb) [file 12913_2019_3882_MOESM4_ESM.pdf]

Manuscript: The effect of a preconception care outreach strategy: The Healthy Pregnancy 4 All study

Additional file 4. Uptake of PCC applications after sending municipal invitation letters diminishes over time.

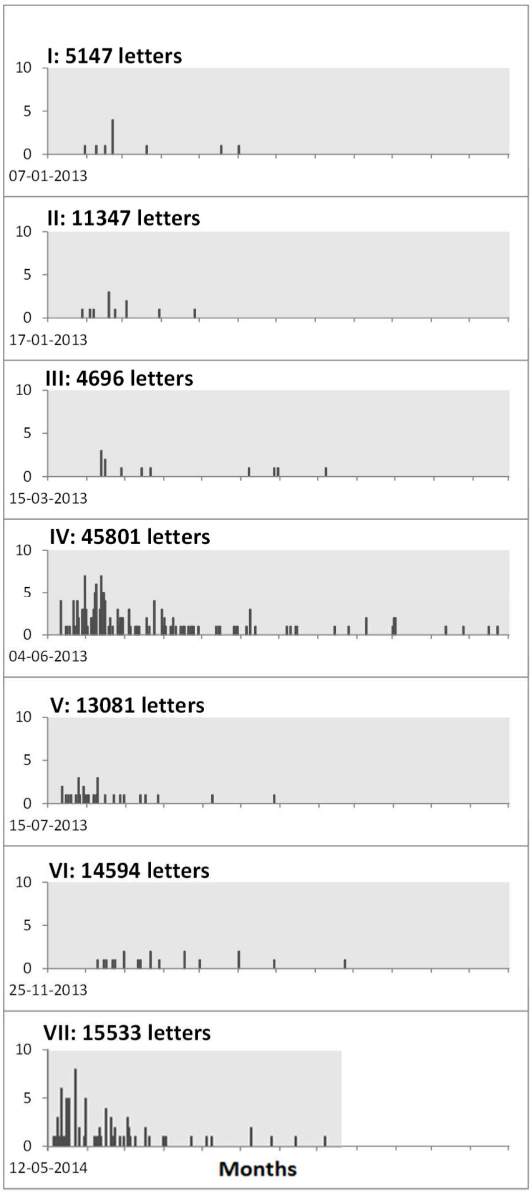

Date and number of municipal letters sent differed per municipality.
Number of PCC applications are shown for a period of a year.*
*Follow up of municipality VII was limited due to ending of the study.
