# Supplementary material for: The effect of a preconception care outreach strategy: the Healthy Pregnancy 4 All study
Source: BMC Health Serv Res. 2019 Jan 23;19:60. doi: 10.1186/s12913-019-3882-y (PMC6343258; doi:10.1186/s12913-019-3882-y)

Additional file 5. Barrier, beliefs and knowledge response per statement (N=237)

Figure 1. Barrier outcome per statement

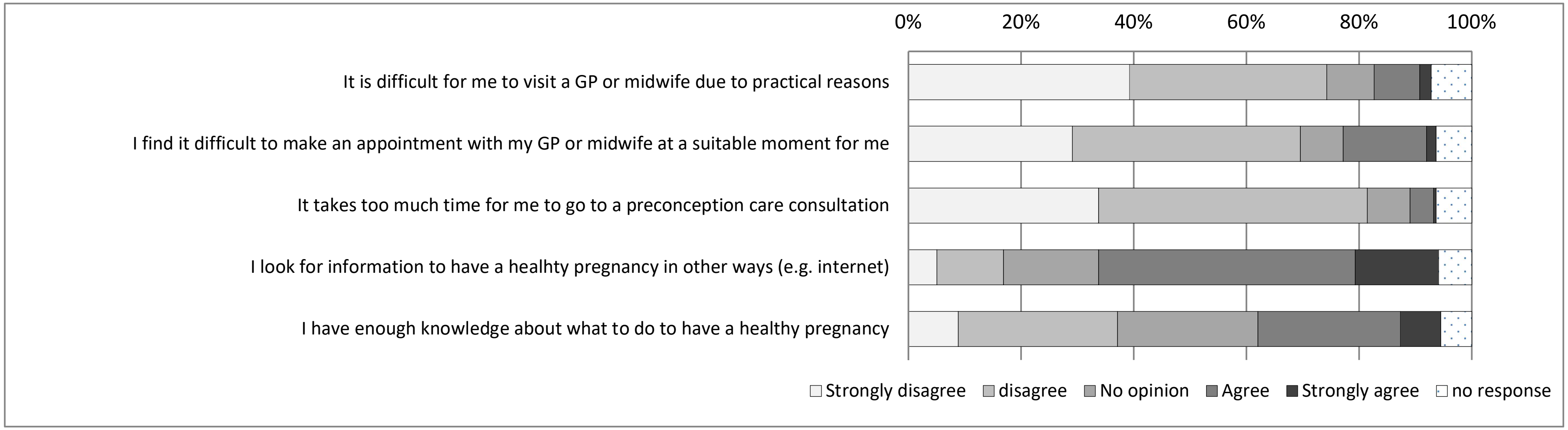

Figure 2. Beliefs outcome per statement

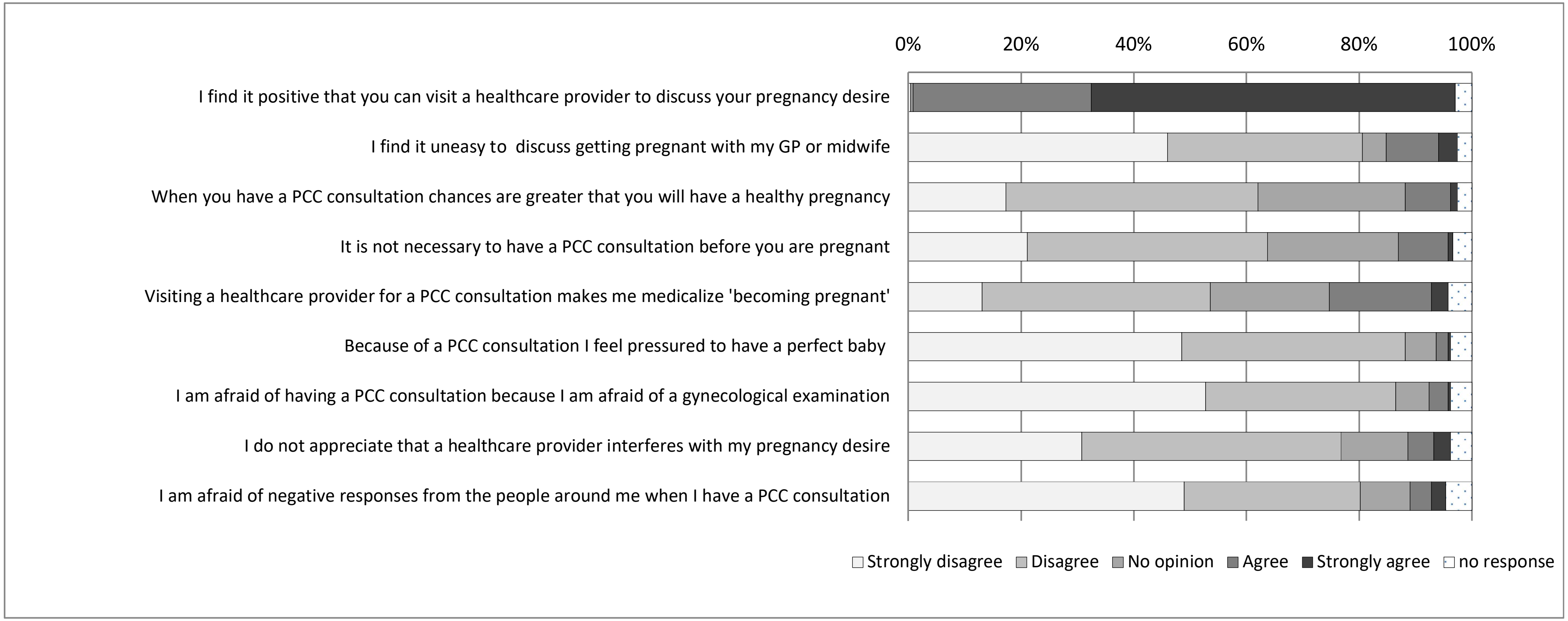

Figure 3. Knowledge outcome per statement

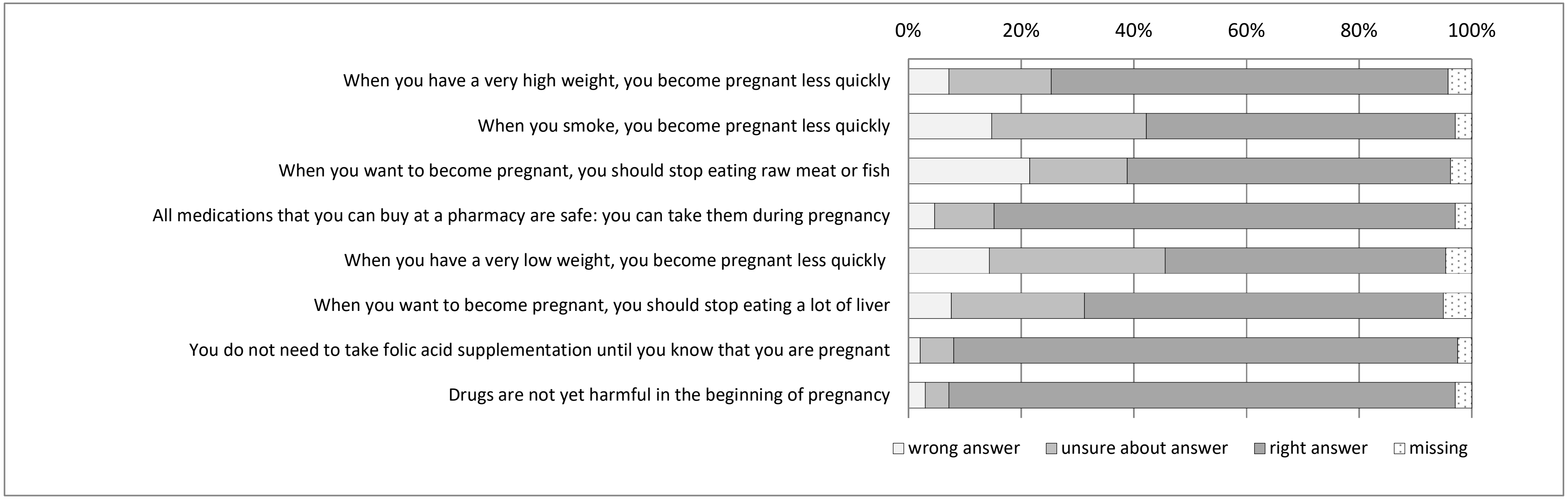

Supplement: Supplementary file 5 — Barrier, beliefs and knowledge response per statement (N = 237). (PDF 352 kb) [file 12913_2019_3882_MOESM5_ESM.pdf]
